# Searching an Effective Therapy for the Coronavirus Pandemic: Do We See Light at the End of the Tunnel?

**DOI:** 10.7759/cureus.7415

**Published:** 2020-03-25

**Authors:** Munish Sharma, Salim Surani

**Affiliations:** 1 Internal Medicine, Corpus Christi Medical Center, Corpus Christi, USA; 2 Internal Medicine, Texas A&M Health Science Center, Bryan, USA; 3 Internal Medicine, University of North Texas, Dallas, USA

**Keywords:** sars-cov-2 (severe acute respiratory syndrome coronavirus -2), coronavirus disease, hydroxychloroquine, azithromycin

## Abstract

Coronavirus disease 2019 (COVID-19) is an emerging infectious disease caused by severe acute respiratory syndrome coronavirus 2 (SARS-CoV-2). First reported at the end of December 2019 as a cause for clusters of pneumonia cases in Wuhan city in China, the rapid spread of this condition was declared a pandemic on March 11, 2020, by the World Health Organization (WHO). Apart from the mortality and morbidity associated with COVID-19, the massive social and financial havoc inflicted by this pandemic has left the entire world pondering if medical science can innovate and curtail the ongoing damage due to SARS-CoV-2. Recent findings of an open-label study that investigated the use of hydroxychloroquine and azithromycin in COVID-19 patients in Marseille, France, has garnered some optimism in scientific quarters and the general public alike in terms of finding a treatment regimen to control the rampant rise of COVID-19. We will discuss the potential off-label therapy and studies as it pertains to COVID-19.

## Introduction and background

Coronaviruses have been long known to be pathogens in both humans and animals. Apart from causing community-acquired coronaviruses, novel forms of coronavirus had been implicated in the outbreak of the severe acute respiratory syndrome (SARS) outbreak in 2003 and the Middle East respiratory syndrome (MERS) in 2012 [1-2]. Towards the end of December 2019, a novel type of coronavirus was identified as the causative agent of a cluster of pneumonia cases in Wuhan city in China. Its rapid spread within China caused an epidemic that is believed to have peaked between late January and early February [3]. Initially referred to as 2019-nCoV, the causative agent is now known as severe acute respiratory syndrome coronavirus 2 (SARS-CoV-2) as recommended by the Coronavirus Study Group of the International Committee on Taxonomy of Viruses [4]. Officially designated as Coronavirus disease 2019 (COVID-19) by World Health Organization (WHO), this novel coronavirus became a pandemic on March 11, 2020 [5]. The total case counts around the world is increasing every day and the latest case counts can be found on the official website of WHO.

SARS-CoV-2 falls in the betacoronavirus subgenus similar to the SARS coronavirus of 2003 and uses the angiotensin-converting enzyme 2 receptor for cellular entry. The closest RNA sequencing has been found to be similar to bat coronaviruses, but it is unknown if there was transmission to humans through an intermediate host or through bats that could be the primary host [6]. In the wake of this pandemic, preventive measures to break the chain of transmission is being adopted. The medical community around the world is also working hard to find effective preventive and curative therapy for those already suffering from this illness. Though difficult, have we started to see some exciting results in our attempt to seek therapy for the COVID-19 disease?

## Review

Revisiting the medications from the SARS and MERS epidemics that have resurfaced in the context of COVID-19

Analysis of the full genomic sequence of the coronavirus that causes COVID-19 has been shown to resemble the SARS coronavirus of 2003 more closely than the Middle Eastern respiratory syndrome (MERS) coronavirus of 2012 [7-8]. After the SARS coronavirus outbreak in 2003, there were multiple attempts to find an effective treatment for the virus. Apart from meticulous supportive care, experts could not recommend specific treatment [9]. During the epidemic in 2003, a vast majority of patients were treated with glucocorticoids and received ribavirin too, but these drugs were not found to have any immediate or long-term beneficial effects [10]. Glucocorticoids were associated with an increased risk of mortality and decelerated the process of viral clearance in MERS coronavirus infection. There was no mortality benefit but rather convincing evidence of harm in the short term and the long run in SARS coronavirus [11]. Staying in lieu of these pieces of evidence, WHO and Centers for Disease Control and Prevention (CDC) have recommended against the use of glucocorticoids in patients with COVID-19 unless there are co-existing compelling indications for its use such as exacerbation of chronic obstructive lung disease or asthma [12].

Ribavirin, a nucleoside analog that has broad antiviral activity, was also used along with glucocorticoids at a high dose via the intravenous and oral routes to combat SARS in 2003. Systematic reviews published in 2005 and 2006 failed to establish any beneficial effects with ribavirin [9-10]. In a prospective observational study that included patients who received ribavirin for 14 days along with glucocorticoids, after initial improvement of fever and symptoms of pneumonia, there was a resurgence of fever in 85% patients within mean 8.9 days while 45% had worsening respiratory symptoms after 8.6 days. Forty-five percent (45%) of patients had newer lesions on radiographs while diarrhea was seen in 73% of patients after 7.5 days of medication use [13]. Despite these findings, there has been a study going on to repurpose the utility of sofosbuvir, a ribonucleic acid (RNA)-dependent RNA polymerase inhibitor used in the treatment of hepatitis C along with ribavirin in COVID-19 patients. Data from sequence analyzing, modeling, and molecular docking from this study have shown the ability of sofosbuvir and ribavirin to tightly bind to the SARS CoV-2 RNA-dependent RNA polymerase, indicating their potential use in the treatment of COVID-19 patients [14]. Further data need to emerge from this study to be considered for clinical use.

Lopinavir and ritonavir, protease inhibitors, were used along with ribavirin and glucocorticoids in an open-label trial during the SARS outbreak in 2003. The treatment group was given lopinavir/ritonavir along with ribavirin and corticosteroids (n=41). It was compared with a control arm with patients treated with ribavirin and glucocorticoids only (n=111). Patients included in the trial did not have acute respiratory distress syndrome due to SARS coronavirus 2003. Severe hypoxemia or death on the 21st day was the composite primary outcome. In the treatment group, 2.4% of patients had severe hypoxemia while there were no deaths in comparison to 22.5% hypoxemia in the control group with 6.3% deaths [15]. Due to the absence of definite treatment guidelines for COVID-19, China International Exchange and Promotive Association for Medical and Health Care (CPAM) issued a guideline in February 2020. They recommended using lopinavir/ritonavir along with interferon-alpha in nebulizer form for antiviral action against SARS-CoV-2. These recommendations were based on weaker evidence [16-17]. A trial of lopinavir/ritonavir conducted in adult patients who were hospitalized with severe COVID-19 failed to show any benefit beyond the standard care available [18]. In this study, 99 patients were treated with lopinavir/ritonavir while 100 patients received standard medical care. There was no difference in time to clinical improvement (hazard ratio 1.24, 95% confidence interval 0.90-1.72), mortality at 28 days (19.2% vs 25%, -5.8% difference with 95% confidence interval -17.3 to 5.7), and decrease in detectable viral load between two arms. Notably, gastrointestinal adverse effects were more common in the lopinavir/ritonavir group that led to 13 patients stopping the medication [18].

Buoyed by its activity against the Ebola virus, remdesivir was also tested and was reportedly found to have some activity against the SARS and MERS coronaviruses [19]. Remdesivir is a nucleoside analog. It is an investigational agent and not available commercially. Its manufacturer, Gilead Sciences, Inc (Foster City, California) has provided it for compassionate use programs on the request of the treating physician and now is requiring institutions to be involved in the randomized trial. Though a clinical trial to determine the efficacy of remdesivir in patients with SARS-CoV-2 infection is ongoing and results may be expected in April 2020, pre-clinical trials have shown significant activity against the coronavirus. The in-vitro study has revealed the potent antiviral activity of remdesivir against SARS-CoV-2 [20-21].

Has the emergence of hydroxychloroquine and chloroquine sparked some optimism?

Chloroquine and hydroxychloroquine, an analog of chloroquine, have been traditionally used as anti-malarial agents and disease-modifying anti-rheumatic drugs (DMARDS) in conditions like lupus and rheumatoid arthritis. Hydroxychloroquine is believed to disrupt vesicle function in the parasite by increasing the pH. It also arrests various erythrocytes stages of the plasmodium to exert its anti-malarial effect. It also inhibits acute phase reactants, rheumatoid factor, and various other enzymes for its immunosuppressive effect [22]. The State Council of China in a news briefing on February 17, 2020, released the first positive experience regarding the use of chloroquine phosphate in patients with COVID-19-associated pneumonia [23]. This was a multicenter clinical trial conducted in 10 different hospitals in China. As per the news briefing by the State Council of China and a further letter published by Gao J et al. on February 19, 2020, more than 100 patients were found to have a lesser exacerbation of pneumonia due to COVID-19, improved radiological findings, better virus-negative conversion, and a shorter course of disease in the chloroquine phosphate treatment arm in comparison to the control group [23]. As per the authors, chloroquine has been recommended to be included in the next edition of official guidelines to be released by the National Health Commission of the People’s Republic of China for the management of pneumonia caused by COVID-19 [23]. The Chinese expert panel has recommended chloroquine 500 mg twice-daily dosing for 10 days [24]. Besides China, several countries, based upon the benefits shown by limited in-vitro studies and anecdotal experiences, are currently using chloroquine or hydroxychloroquine in patients with COVID-19 pneumonia.

On March 17, 2020, findings were released from an open-label study that investigated the use of hydroxychloroquine and azithromycin in hospitalized patients with COVID-19. This study was conducted at the Mediterranee Infection University Hospital Institute in Marseille, France [25]. Only patients above the age of 12 years were included. The polymerase chain reaction (PCR) technique was used to detect SARS-CoV-2 from the nasopharyngeal samples of the patients on admission. The study enrolled 36 out of 42 patients meeting the inclusion criteria. Twenty-six patients received hydroxychloroquine while 16 patients were the control. It has been reported that six patients were lost to follow-up due to the early cessation of treatment. This study included asymptomatic patients (16.7%), patients with upper respiratory tract symptoms (URTI) 61.1%, and lower respiratory tract symptoms (LRTI) 22.2%. The authors have confirmed that all cases of LRTI had pneumonia confirmed on radiological findings of Computed Tomography (CT) chest. All the demographic factors in the treatment and control arms were well-matched except that the average age of hydroxychloroquine-receiving patients was 51.2 years versus 37.3 years in the control group. All 20 patients in the treatment arm were administered 200 mg of oral hydroxychloroquine three times a day for 10 days. In the control arm, six patients also received azithromycin at a dose of 500 mg on the first day followed by 250 mg daily for four days to prevent secondary bacterial infection. The primary endpoint was virological clearance on the sixth day of the initiation of treatment. The results of this study revealed that 70% of hydroxychloroquine-treated patients were free of the virus as compared to only 12.5% of patients in the control group. Interestingly, all six patients (100%) treated with hydroxychloroquine and azithromycin were cured. One patient who received hydroxychloroquine only remained PCR-positive on the sixth day and was able to get rid of the virus on the ninth day after the addition of azithromycin on the eighth day [25].

We also personally opine that the results of this study are promising especially in the current context of this pandemic where the entire world is struggling to find a definitive treatment. Apart from a cure for the existing patients, this study opens avenues for further studies geared towards finding a prophylactic regimen also that can stop the emergence of new cases and limit the burden of this pandemic. However, we do have to acknowledge that this study is limited due to its smaller sample size, restricted ethnic and geographic population, and very short-term follow-up; it also raises further queries and concerns regarding the six patients that dropped out of the study. Results look more promising in patients who received a combination of hydroxychloroquine and azithromycin but whether the combination of these two medications leads to potential cardiac-elated adverse events, such as ventricular arrhythmias, mainly due to the risk of QT prolongation, is something that needs to be addressed in future studies. Another question this study raises is the possibility of the existence of certain resistant strains of SARS-CoV-2 that did not respond to hydroxychloroquine treatment.

In the United States as well, clinical trials involving hydroxychloroquine are being planned for not only the treatment of mild, moderate, and severe COVID-19 but also for pre and postexposure prophylaxis [26].

Quest for a vaccine

There is currently no vaccine that has been approved for pre-exposure or postexposure prophylaxis. There are various clinical trials that are being conducted in the United States and in other countries for postexposure prophylaxis [27]. In the United States, the first vaccine that is being evaluated for the prevention of COVID-19 is using a technique that would promote the expression of viral protein, which can potentially induce a response from the immune apparatus of the host [28]. The entire world is looking forward to a breakthrough in terms of vaccines but we have to wait patiently for now.

## Conclusions

In the wake of the pandemic caused by COVID-19, the medical and scientific community around the world has been racing against time to find a cure for SARS-CoV-2. The sudden onset and unprecedented surge of this virus have instilled an urgency to seek preventive and therapeutic options for COVID-19. The recent outpouring of data from the rather smaller studies and anecdotal experiences cannot be considered a major breakthrough but may be looked at as a silver lining. Further, bigger studies are needed to be started immediately to consolidate the data obtained from these smaller studies. We must make a careful choice while designing larger studies based on the preliminary data from smaller studies, as we might not only risk treating patients with therapy that may not ultimately prove to be effective but may also miss the opportunity to pursue better options.
